# The radiological diagnosis of pregnancy associated venous thromboembolism: a review of current research

**DOI:** 10.3389/fmed.2024.1394012

**Published:** 2024-08-15

**Authors:** Di Yang, Li Wang

**Affiliations:** Changzhou Maternity and Child Health Care Hospital, Changzhou Medical Center, Nanjing Medical University (Changzhou Maternity and Child Health Care Hospital), Changzhou, China

**Keywords:** venous thromboembolism, pregnancy, deep vein thrombosis, pulmonary embolism, radiological diagnosis

## Abstract

One of the main causes of unfavorable pregnancy outcomes in expectant mothers is pregnancy-associated venous thromboembolism. Although pregnancy-related venous thromboembolism does not always manifest obvious clinical symptoms and lacks a comprehensive standard risk assessment and prediction system as well as simple and effective laboratory testing techniques, timely and accurate diagnosis can still help reduce the probability of adverse pregnancy outcomes. To aid in the early detection, diagnosis, and treatment of pregnancy- associated venous thromboembolism, we attempt to provide an overview of the radiological diagnostic techniques for various forms of the condition.

## 1 Introduction

It is commonly known that one of the main factors contributing to pregnant women’s higher complications and fatality rates is pregnancy-associated venous thromboembolism (PA-VTE). The disease’s occurrence has increased due to the specificity of the physiological status of women who are pregnant or just gave birth (Venous stagnation, hypercoagulable state of blood, and pelvic vascular endothelial injury caused by childbirth, etc.), as well as the absence of a thorough, standardized method for assessing PA-VTE risk (1). Existing PA-VTE risk assessment models typically focus only on VTE risk factors validated by large-scale studies (2, 3), yet using these factors to assess PA-VTE risk often proves inadequate. The physiological and pathological conditions of pregnant and postpartum women differ from those of non-pregnant individuals, and their physical and mental states undergo dynamic changes throughout pregnancy. Existing PA-VTE risk assessment models often employ static risk factors, such as age, gender, body mass index (BMI), past medical history, genetic history, lifestyle and so on. Thus, while data show that the application of these models has reduced maternal deaths to some extent, their predictive accuracy and precision still require enhancement. Some high-risk pregnant women may be underestimating their risk of developing PA-VTE, potentially leading to irreversible severe outcomes, while some low-risk women may be overestimating their risk, resulting in excessive antithrombotic therapy. According to a study conducted in more than 100 national hospitals, the rate of maternal death attributable to PA-VTE was 3.2% (4). The percentage of elderly pregnant women and *in vitro* fertilization (IVF) has increased dramatically due to the growing demands placed on today’s populations as well as scientific and technological advancements. The incidence and negative outcomes of PA-VTE in assisted reproductive technology pregnancies have grown in comparison to natural pregnancies, regardless of the technology’s success (5). According to studies by Zhao et al. (6), the majority of pregnant women who encounter PA-VTE do not receive early screening and prevention in prenatal care for a variety of reasons. There are still signs to look out for and treatment options that may be taken to lessen the likelihood of an unfavorable prognosis, even if the incidence rate of PA-VTE is substantial and causes more unfavorable obstetric outcomes. PA-VTE has also drawn more interest in recent years. Studies that are pertinent to the topic have demonstrated that prompt diagnosis and sensible intervention techniques can aid in the treatment of PA-VTE and even stop it from developing, which lowers the risk of unfavorable obstetric events. An overview of the development of PA-VTE diagnostic research is given in this article.

## 2 The concept of venous thromboembolism

The improper coagulation of blood in a vein that leads to the creation of a venous thrombus is known as venous thromboembolism (VTE). The detached thrombus, also known as a thrombus, travels into other organs with the flow of venous blood. This obstructs the blood arteries in the corresponding organ and prevents normal blood circulation, resulting in the occurrence of thrombotic diseases which may have no exhibit overt clinical symptoms. Pulmonary embolism (PE) and deep vein thrombosis (DVT) are the two main categories under which VTE falls (7). DVT is the term for abnormal blood coagulation in deep veins that causes embolism by obstructing the venous return channel at the coagulation site. Although research has indicated that DVT frequently happens in the deep veins of the lower limbs, it can also occasionally occur in other venous systems, Harnik et al. documented cases of mesenteric venous thrombosis, for example (8). PE is the term used when liquid, solid, or gas components obstruct one or more pulmonary arteries. PE is primarily brought on by blood clots that originate in the deep veins of the legs or pelvis, break off, and travel to the lungs via the venous return (9, 10). Studies have demonstrated that women who ultimately undergo cesarean section due to factors such as unsuitability for natural childbirth or personal choice are more likely to develop PE, and that PE is more likely to directly result in maternal mortality when compared to DVT (11).

## 3 The etiology of venous thromboembolism

Currently, endothelial damage, blood in a hypercoagulable state, and venous blood stasis are the three recognized components of thrombosis, sometimes known as the Virchow triad. Pregnant women’s blood physiological changes perfectly match the aforementioned risk factors (12). According to available data, pregnant women’s levels of coagulation factors significantly alter during pregnancy. In contrast to non-pregnant situations, the concentrations of coagulation factors V, VII, VIII, IX, X, XII, and vascular hemophilia factor increased, and there was a considerable decrease in the activity of protein S, which has physiological anticoagulant effects. The body’s fibrinolytic activity is also momentarily impacted during pregnancy, but these hematological indicators swiftly restore to their pre-pregnancy levels following delivery (13). Pregnant women who experience any of these circumstances are likely to be hypercoagulable during pregnancy. According to research by James et al. (14), pregnant women can be somewhat protected by this hypercoagulable state, which can also lessen or prevent heavy bleeding during childbirth or miscarriage. However, pregnant women are also more likely to develop VTE than non-pregnant women, with the risk being more than four times higher (15). Multiple pregnancies, advanced age, cesarean sections, and other variables have also been linked to an increased chance of developing PA-VTE, according to a significant number of previous research (16). Taller pregnant women are more likely than the general population to have venous thrombosis during pregnancy because of studies that have demonstrated that they have more venous valves as a result of their bigger venous surface area (17, 18).

## 4 Diagnosis of venous thromboembolism

According to Zhang et al.’s research, the riskiest time to develop PA-VTE is one week following delivery (19). The diagnosis of PA-VTE is made more challenging by the findings of Elgendy et al.’s study, which showed that many clinical symptoms and indicators of the condition are comparable to physiological changes that occur during pregnancy, such as lower limb edema, dyspnea, and tachycardia (20). As a result, in clinical practice, diagnosing PA-VTE requires combining patient complaints with imaging tests. However, because pregnant and postpartum women are unique, there are additional factors that need to be taken into account when performing imaging examinations. These include whether the examination will have an impact on fetal growth and development, its detection rate, and how convenient it will be for both the examination physicians and the pregnant and postpartum patients.

### 4.1 Diagnosis of DVT

The left lower limb accounts for the great majority of DVT cases during pregnancy, which may be connected to the anatomical and physiological location of the left common iliac vein. It is situated in the space between the fifth lumbar vertebra and the right common iliac artery. It is narrower than the right common iliac vein, has a sluggish blood flow velocity, and is prone to vortex formation, all of which can increase the risk of venous thrombosis. Color Doppler ultrasonography (CUS) testing should be done initially when clinical physicians suspect DVT in pregnant women presenting with clinical symptoms of inguinal pain, unilateral leg pain, or edema (21). Women who are pregnant or recently gave birth typically accept CUS more readily because it is a quick, affordable, environmentally friendly, radiation-free, repeatable test that can be completed in a short amount of time. CUS examination is considered a traditional diagnostic approach for DVT, with a study indicating that the diagnosis rate of DVT through CUS is roughly 98.7% (22). Despite the fact that multi-slice CT venography (MSCTV) and contrast-enhanced venography (CV) offer incredibly high diagnosis rates and a sharper depiction of venous orientation. However, these tests are typically invasive and need for the use of radioactively infused contrast chemicals. According to certain criteria, CUS tests, which have a high sensitivity and specificity, can generally substitute CV and MSCTV examinations in the diagnosis of DVT (23). A CUS examination is sufficient for the majority of pregnant women suspected of having DVT; nevertheless, some pregnant women show normal results on the CUS examination but continue to have comparable clinical symptoms. In order to rule out or diagnose DVT in these groups, a UK study recommends that either a magnetic resonance imaging (MRI) or a CUS test be done for a week (21). Additionally, MRI examination does not necessitate the use of radioactive contrast agents or exposure to ionizing radiation during the examination process. In comparison to CUS examination, it can achieve high-quality imaging with computer-aided assistance and better visualize the vena cava and pelvic veins (24). The study by Torkzad et al. (25) also suggests that MRI examination typically has a higher detection rate in diagnosing DVT of the pelvic vein, and even within the population enrolled in this research, a patient with a thrombus that had migrated to the inferior vena cava only received positive results through MRI examination. A case report by Dronkers et al. (26) on iliac vein thrombosis further supports the importance of MRI in diagnosing DVT of the pelvic vein.

### 4.2 Diagnosis of PE

The well-known trinity of pulmonary embolism (PE) symptoms—chest discomfort, hemoptysis, and dyspnea—are frequently utilized as early clinical indicators to diagnose PE. However, this is frequently hampered by typical physiological reactions that occur during pregnancy, such as physiological dyspnea and chest discomfort (20, 27). According to a study conducted in the US, PE affects about 370,000 people yearly and may eventually result in 60,000–100,000 fatalities (10). Clinical physicians should exercise caution when dealing with the aforementioned symptoms and have the necessary examinations as soon as feasible to rule out PE because of the high mortality risk of PE (28). Evidence-based diagnostic recommendations for PE have been developed by the American Thoracic Society and the American College of Obstetricians and Gynecologists (29). According to the guidelines, patients should initially get a chest X-ray examination if they have matching concerns. It is commonly recognized that chest X-ray exams are inexpensive and simple to perform, making them a popular choice for radiation exams in clinical settings. The positive diagnosis rate of a chest X-ray was only 59% in a research with 436 confirmed PE patients (30), and the limited specificity of this diagnostic technique means that it is not as frequently employed in clinical practice for PE diagnosis or differentiation. This examination technique is frequently used by clinicians as a PE screening tool. It is advised to have a ventilation perfusion (V/Q) examination if a chest X-ray is negative for pregnant and postpartum women who continue to report pertinent clinical symptoms in order to prevent missed diagnosis. While radioactive contrast chemicals, like iodohexanol, are necessary for pulmonary V/Q assessment, pregnant women and fetuses may be exposed to radioactive nuclides as a result of this procedure (31, 32). Nonetheless, research has demonstrated that the dose of radioactive isotopes utilized for lung V/Q evaluation is far less than the dose that may affect the fetus and mother (33). Therefore, we still advise that patients with clinical symptoms but a negative chest X-ray obtain further lung V/Q testing in order to diagnosis or rule out PE, taking into account the benefits and drawbacks of doing so. Clinicians also need to consider whether the examination may lead to over-testing for patients, resulting in over-treatment of small blood clots that do not yet require treatment, as these treatment procedures are bound to pose potential risks to pregnant women and foetuses. Simultaneously, research has indicated that lung perfusion scanning can be carried out prior to lung V/Q assessment. In addition to lowering the amount of radioactive isotopes that the mother and fetus are exposed to as a result of this examination, performing lung ventilation scanning again is not necessary if the examination results are normal (34). Clinical physicians must do Computed Tomography Pulmonary Angiography (CTPA) on patients exhibiting clinical symptoms if their chest X-ray scan yields good results. Previous research has demonstrated that CTPA is a highly sensitive and negative predictive value test for PE diagnosis. As a result, CTPA is frequently utilized as the “gold standard” for PE diagnosis in real-world clinical settings (35). However, some research also suggests that lung V/Q test, which is appropriate for ruling out PE, has the same effect as CTPA (36). The choice between X-ray examination, V/Q scan, or CTPA in clinical practice may depend more on the experience of physicians and the individual preferences of patients and their families because there is a dearth of evidence on whether examinations offer clear advantages.

## 5 Future outlook

The aforementioned information indicates that PA-VTE, which has a variety of etiological variables and physiological traits specific to pregnant women, is a key contributor to the rise in the global rate of maternal death. Clinical practitioners have many constraints when choosing critical imaging examination techniques during diagnosis because of the physiological characteristics of maternal women and fetal. Due to physiological changes, such as uterine enlargement and compression of blood vessels during pregnancy, pregnant women often experience symptoms like lower limb swelling and pain, which can interfere with the initial diagnosis of PA-VTE based on clinical symptoms. While existing diagnostic imaging techniques offer a reliable basis for the diagnosis of PA-VTE to a certain extent, they still have several limitations. For instance, CUS lacks sensitivity for detecting small blood clots in blood vessels, and CTPA is an invasive procedure that requires the use of radioactive isotopes, making it unsuitable and undesirable for all pregnant and postpartum women to undergo such examinations.

However, with the patient’s informed agreement, we can still carefully carry out some important imaging examinations that will not significantly affect the mother and fetus after carefully weighing the benefits and disadvantages. This is made possible by the advancement of medical technology. The diagnosis of PA-VTE can also benefit from laboratory testing, such as measuring blood levels of D-dimer, which indicates coagulation status. Nonetheless, it’s critical to establish particular diagnostic standards catered to the requirements of pregnant women, considering the physiological rise in D-dimer levels during pregnancy. In order to better protect pregnant women and fetuses, we aspire to find more environmentally friendly, secure, and highly accurate examination techniques in the future. In order to decrease false positives, clinical physicians can collaborate with laboratory specialists to improve the specificity of hematological monitoring markers. They can also work with radiologists to explore optical scanning diagnostic techniques, like Optical coherence tomography (OCT), with the goal of improving the safest methods for detecting small and elusive venous thrombi. To create PA-VTE diagnostic criteria and risk assessment prediction models specifically for expectant mothers, obstetricians and gynecologists can work with the departments of hematology, laboratory, and radiology.
